# Altered Cardiac Repolarization in Association with Air Pollution and Air Temperature among Myocardial Infarction Survivors

**DOI:** 10.1289/ehp.1001995

**Published:** 2010-09-15

**Authors:** Regina Hampel, Alexandra Schneider, Irene Brüske, Wojciech Zareba, Josef Cyrys, Regina Rückerl, Susanne Breitner, Harald Korb, Jordi Sunyer, H.-Erich Wichmann, Annette Peters

**Affiliations:** 1 Helmholtz Zentrum München—German Research Center for Environmental Health, Institute of Epidemiology II, Neuherberg, Germany;; 2 Cardiology Division, University of Rochester Medical Center, Rochester, New York, USA;; 3 University of Augsburg, Environmental Science Center, Augsburg, Germany;; 4 Philips HeartCare Telemedicine Services GmbH, Duesseldorf, Germany;; 5 Center for Environmental Epidemiological Research, Municipal Institute for Medical Research, Universitat Pompeu Fabra, Barcelona, Spain;; 6 Department of Epidemiology, Ludwig-Maximilians-University of Munich, Munich, Germany

**Keywords:** air pollution, air temperature, epidemiology, myocardial infarction, panel study, repolarization

## Abstract

**Background:**

Epidemiological studies have shown that ambient particulate matter (PM) and changes in air temperature are associated with increased cardiopulmonary events.

**Objective:**

We hypothesized that patients with previous myocardial infarction (MI) experience changes in heart rate (HR) and repolarization parameters, such as Bazett-corrected QT interval (QTc), and T-wave amplitude (Tamp), in association with increases in air pollution and temperature changes.

**Methods:**

Between May 2003 and February 2004, 67 MI survivors from the Augsburg KORA-MI registry repeatedly sent 16 sec electrocardiograms (ECGs) with a personal transmitter (Viapac) via telephone to the Philips Monitoring Center, where ECG parameters were immediately analyzed. Meteorological data and air pollutants were acquired from fixed monitoring sites on an hourly basis. Additive mixed models were used for analysis. Effect modification by patient characteristics was investigated.

**Results:**

The analysis of the 1,745 ECGs revealed an increased HR associated with interquartile range (IQR) increases in PM levels among participants not using beta-adrenergic receptor blockers and among those with body mass index ≥ 30 kg/m^2^. We observed a 24- to 47-hr lagged QTc prolongation [0.5% change (95% confidence interval, 0.0–1.0%)] in association with IQR increases in levels of PM ≤ 2.5 μm in aerodynamic diameter, especially in patients with one [0.6% (0.1–1.0%)] or two [1.2% (0.4–2.1%)] minor alleles of the nuclear factor (erythroid-derived 2)-like 2 (*NFE2L2*) single-nucleotide polymorphism rs2364725. Positive immediate (0–23 hr) and inverse delayed (48–71 hr up to 96–119 hr) associations were evident between PM and Tamp. We detected an inverse U-shaped association between temperature and Tamp, with a maximum Tamp at 5°C.

**Conclusions:**

Increased air pollution levels and temperature changes may lead to changes in HR and repolarization parameters that may be precursors of cardiac problems.

Numerous studies have shown that elevated ambient air pollutants and changes in air temperature are associated with increases in hospital admissions and mortality due to cardiovascular events (Analitis et al. 2008; Pope and Dockery 2006). The effects of air pollution on heart rate (HR) and heart rate variability (HRV) have been studied more extensively since the initial publications by Pope et al. (1999), Peters et al. (1999), and Gold et al. (2000). For example, researchers reported a reduced HRV in susceptible participants such as senior adults (Luttmann-Gibson et al. 2006) and patients with coronary artery disease (CAD) (Timonen et al. 2006). Increased levels of air pollution have been shown to enhance the risk for ST-segment depression (Pekkanen et al. 2002) and arrhythmia (Berger et al. 2006). It is hypothesized that the observed associations between HR and HRV and air pollutants are a consequence of the activation of the autonomic nervous system or a direct affection of the electric system of the heart (Pope and Dockery 2006). Little is known about the influence of temperature on HR and HRV. Drops in temperature may activate the sympathetic nervous system via stimulation of cold receptors in the skin, which may result in increased catecholamine levels. The consequences are vasoconstriction and increased blood pressure (Alpérovitch et al. 2009; Pääkkönen and Leppaluoto 2002).

Potential mechanisms of the influence of air pollutants and temperature on repolarization have received less attention. Some researchers have hypothesized that a prolonged QT interval and T-wave abnormalities might trigger the onset of arrhythmias (Roden 2008) and increase the risk for coronary deaths (Greenland et al. 2003). Only a few studies have investigated the relationship between elevated levels of particulate matter (PM) air pollution and repolarization thus far (Ghelfi et al. 2008; Henneberger et al. 2005; Zareba et al. 2009), and little is known about the temperature influence on these parameters.

The main objective of our study was to evaluate the influence of air pollutants and air temperature on repeated measurements of HR and repolarization parameters, such as Bazett-corrected QT interval (QTc) (Bednar et al. 2001) and T-wave amplitude (Tamp).

Because specific single-nucleotide polymorphisms (SNPs) have been reported to modulate the QT interval (Pfeufer et al. 2009), we examined modifications of the association between air pollution and electrocardiogram (ECG) parameters by SNPs involved in detoxification pathways.

## Materials and Methods

### Study population

A panel study of nonsmoking myocardial infarction (MI) survivors was conducted in Augsburg, Germany, between 30 May 2003 and 1 February 2004, as a substudy of the AIRGENE (Air Pollution and Inflammatory Response in Myocardial Infarction Survivors: Gene-Environment Interaction in a High Risk Group) study. Only persons who had survived an MI between 3 months and 6 years before entry into the study were included. All methods used in the study center were conducted according to common standard operating procedures (Peters et al. 2007). All participants gave written informed consent, and the study protocol was approved by the German ethics commission (Bayerische Landesaerztekammer). During a clinical examination, a baseline questionnaire (Peters et al. 2007) was administered regarding health status, self-report of current medication intake, and smoking history; blood pressure and body mass index (BMI) also were measured. A more detailed description of the patient recruitment and the study panel can be found elsewhere (Peters et al. 2007).

### Clinical measurements

At the beginning of the study, the participants were asked whether they wanted to transmit an ECG only in case of cardiac symptoms or daily at the same time during the whole study period. Participants were invited to call the Philips Monitoring Center in Düsseldorf, Germany, at any time and transmit an ECG via telephone (land line or mobile). For this purpose, a participant had to fix a 12-lead personal ECG transmitter (Philips Viapac, Philips Telemedicine and Healthcare Services, Dusseldorf, Germany; size ~ 13 × 7 × 5 cm; weight, 35 g) with a belt on the chest, which placed the electrodes automatically in the correct position (Mampuya 2002; Mischke et al. 2005). The medical staff of the monitoring center questioned the individuals about cardiac symptoms and whether they had been in traffic or had been exposed to great physical strain or anger 2 hr before the transmission. The staff, which comprised cardiologists and trained medical professionals, immediately evaluated the ECG manually.

Each participant repeatedly transmitted 16 sec ECGs to the monitoring center. If an individual sent two ECGs within 30 min, we excluded the first measurement assuming that the participant thought that the first transmission was incorrect and hence transmitted a second one. If there were several transmissions per day and per patient, only the first one (in case of no second transmission within 30 min) was included in the analysis. ECG measurements with a pacemaker rhythm, frequent ectopic ventricular beats, or atrial fibrillation were excluded. One individual with an internal defibrillator was kept in the study because he had a normal heart rhythm without conduction abnormalities during ECG transmissions.

The outcomes of interest were HR, QTc, Tamp, PQ interval, ST-segment changes, and ventricular and supraventricular ectopic beats. RR-, QT-, and PQ-interval durations were measured manually in lead II. We chose lead II for QT measurements because in clinical conditions this lead is considered representative of the overall electrical forces of the heart. For Tamp, we used ECG leads I, II, and V1–V6, and the median value from those eight original leads was taken for each cardiac cycle. For ST-segment leads LII (inferior wall), V2 (anterior wall), and V5 (lateral wall) were used, respectively, and the ST segment was the median value over each 16-sec period.

### Genotyping

DNA was extracted from ethylenediaminetetraacetic acid anticoagulated blood using a salting out procedure. In our analysis, we used 12 SNPs located in four genes involved in detoxification pathways. The SNPs were rs10183914, rs1806649, rs1962142, and rs2364725 in the nuclear factor (erythroid-derived 2)-like 2 (*NFE2L2*) gene; rs1048942 and rs2606345 in the cytochrome P450 family 1 member A1 (*CYP1A1*) gene; rs1799945 and rs1800562 in the hemochromatosis (*HFE*) gene; rs1695, rs6591256, and rs4891 in the glutathione *S*-transferase pi (*GSTP1*) gene; and a deletion in glutathione *S*-transferase mu 1 (*GSTM1*). SNPs with a minor allele frequency (MAF) < 5% were excluded. Each SNP was tested for deviations from Hardy-Weinberg equilibrium (HWE). For the analyses, SNPs were coded linearly, counting the number of minor alleles. For frequencies of rare homozygote genotypes < 5%, the hetero- and minor homozygote genotypes were combined into one group.

### Air pollution and meteorological data

Air pollution data from fixed monitoring sites representing urban background concentrations were collected according to standard procedures (Rückerl et al. 2007). Hourly means of carbon monoxide (CO), nitrogen dioxide (NO_2_), and PM with an aerodynamic diameter ≤ 10 μm or 2.5 μm (PM_10_, PM_2.5_) were available. Particle number concentrations (PNC) were obtained as a proxy for ultrafine particles (UFP) with an aerodynamic diameter of 0.01–0.1 μm. Additionally, we computed coarse particles (PM_10–2.5_) as the difference between PM_10_ and PM_2.5_. Meteorological variables (air temperature, relative humidity, barometric pressure) were obtained through a monitoring system operated by the Bavarian Environment Agency (Bayerisches Landesamt für Umwelt).

For each person and ECG transmission, we determined average exposures for 0–23 hr before ECG transmission and for up to four 24-hr periods before (24–47, 48–71, 72–95, 96–119 hr) if more than two-thirds of the hourly air pollution or meteorological measurements were available for a given period. Additionally, we calculated mean exposures during the 5 days (120 hr) before ECG transmissions. Missing data on the aggregate level were replaced using a formula adapted from the APHEA (Air Pollution and Health: A European Approach) method (Berglind et al. 2009).

### Statistical analysis

The longitudinal data were analyzed with the SAS statistical package (version 9.1; SAS Institute Inc., Cary, NC, USA) using additive mixed models with a random patient effect. To account for the dependencies between the inordinate repeated measurements, we assumed a spatial covariance structure. The elements of this covariance matrix decline as the elapsed time between two measurements increases.

For analysis of air pollution effects, we identified confounders for each ECG parameter separately. Potential confounders were long-term time trend, day of the week, temperature, relative humidity, and barometric pressure. The possible lags for meteorology were defined as 0–23, 24–47, 48–71, and 72–95 hr before ECG transmission. The confounders were modeled linearly or as penalized splines (P-splines) to allow for nonlinear relationships. The lag and shape that minimized the Akaike information criterion (AIC) was selected. If a confounder was included as a P-spline, we checked whether a polynomial led to a smaller AIC. Barometric pressure and day of the week were selected only if model fit was improved. For the analysis of temperature effects, only trend and relative humidity with a corresponding lag to the analyzed temperature lag were included as confounders.

After assessing the confounder model, single air pollution or temperature lags were added, and the effects were estimated linearly.

### SNP selection

The influence of the selected SNPs on the mean or variability of the ECG parameters was estimated separately. In order to assess the association between an SNP and variability, mixed models with two different covariance structures were calculated. As described above, the first model used a spatial covariance matrix with blocks identical for each participant, but the second model included a matrix with three different blocks identical only for patients with the same genotype. If the likelihood ratio test revealed a significant difference between the two models, we assumed that the variability of the ECG parameter differed between the genetic groups. For SNP selection, the alpha-level was corrected for the number of independent tests following a modified Bonferroni procedure (Li and Ji 2005).

### Effect modification

Interaction variables were added to the model in order to estimate the air pollution or temperature effects of the corresponding subgroups. For air pollution, separately included interaction variables were age (age < 60 vs. ≥ 60 years), BMI (< 30 vs. ≥ 30 kg/m^2^), beta-adrenergic receptor blockers (intake vs. no intake), sex (women vs. men), season (summer April–September vs. winter October–March), smoking status (ex-smoker vs. never-smoker), being in traffic 2 hr before the ECG transmission (yes vs. no), and SNPs with a significant influence on the mean or variability of at least one ECG parameter. For temperature, we assessed interactions with age, BMI, intake of beta blockers, sex, and season. To investigate possible interaction effects between exposure variables, we calculated smooth interaction functions of air pollution and temperature using R version 2.5.0 (R Foundation for Statistical Computing 2008). For this purpose, we used the exposure lags that showed the strongest associations with the ECG parameters in the performed main analyses that included exposure variables separately as fixed effects in mixed models.

### Sensitivity analyses

In order to check the robustness of our models, we performed several sensitivity analyses. For air pollution and temperature effect estimation, we excluded ECGs with ectopic beats or ECGs transmitted when participants indicated they were having cardiac problems, respectively. In a further sensitivity analysis, individuals with QRS intervals > 120 msec in at least two ECGs were excluded. Furthermore, we performed an alternative confounder model including patient characteristics such as blood pressure, BMI, sex, smoking status, or physical activity if this minimized the AIC. Afterward, the meteorological confounders were selected as described above. Because PNC was not measured for several weeks during the study period, we additionally estimated the association between PM or gaseous pollutants and ECG parameters only on days with existing PNC measurements.

For temperature effect estimation we added barometric pressure, PM_10_, or PM_2.5_ separately to the original confounder model. Barometric pressure was included with the same lag as the analyzed temperature lag. PM was included either with the same lag as temperature or with the lag exhibiting the largest association with the outcome in the air pollution analysis. The shape (linear, smooth, polynomial) of barometric pressure and air pollutants that minimized AIC was chosen, respectively. Additionally, temperature effects were estimated as P-splines.

## Results

### Study population

A total of 75 individuals participated in the substudy and received a Viapac system from Philips. Two patients were ineligible because they had not had a previous MI according to the official criteria; we excluded five patients because they did not transmit any ECG or transmitted only one ECG to the monitoring center. We excluded individual ECG measurements for the following reasons: abnormal pacemaker rhythm or ventricular trigeminus (51 ECGs from 3 patients), atrial fibrillation (37 ECGs from 5 patients), transmission while not in Augsburg (14 ECGs from 10 patients) or from an unknown location (2 ECGs from 2 patients), second ECG transmitted within 30 min (3 ECGs from 3 patients), and second ECG transmission within 1 day but not within 30 min of another transmission (14 ECGs from 9 patients). We excluded one patient because only one eligible ECG was available after we excluded a second ECG due to atrial fibrillation. Therefore, 1,745 ECGs from 67 patients were available for the analyses. Only 3 of 21 persons who agreed to transmit ECGs on a daily basis did so; 46 persons declared intention to transmit ECGs only in case of cardiac problems. However, in both groups only 16 ECGs (2%) were sent due to heart trouble, respectively. The participants transmitted on average 26 ECGs (range, 3–178 ECGs) and experienced an MI on average 2.2 years (range, 0.6–3.5 years) before study entry. The age of the participants ranged from 40 to 76 years. In Table 1, we provide further clinical patient characteristics.

### Clinical measurements

Table 2 describes the ECG parameters analyzed. HR, QTc, Tamp, and PQ interval were not correlated according to the median of patient-specific Spearman correlation coefficients (data not shown). We did not evaluate ST-segment changes because they were available for only 72 ECGs (4%). We did not analyze ectopic beats because only 76 ECGs (4%) from 19 participants and 31 ECGs (2%) from 12 participants exhibited ventricular and supraventricular ectopic beats, respectively.

### Air pollution and meteorological data

During the study period the 24-hr averages ± SD were 33.4 ± 13.2 μg/m^3^ for PM_10_, 15.8 ± 7.7 μg/m^3^ for PM_10–2.5_, 17.7 ± 6.2 μg/m^3^ for PM_2.5_, 11,809 ± 6,253/cm^3^ for PNC, 40.7 ± 10.8 μg/m^3^ for NO_2_, 0.6 ± 0.2 mg/m^3^ for CO, 10.8 ± 9.9°C for air temperature, 69.1 ± 14.5% for relative humidity, and 1018.2 ± 6.9 hPa for barometric pressure [for additional information, see Supplemental Material, Table 1 (doi:10.1289/ehp.1001995)]. Spearman correlation coefficients among the three PM measurements and between CO and PNC, PM_10–2.5_ and NO_2_, and temperature and relative humidity revealed moderate or strong correlations (|*r*| > 0.5). The remaining air pollutants and meteorological covariates were not correlated. Supplemental Material, Figure 1 (doi:10.1289/ehp.1001995), depicts daily 24-hr averages of PM_2.5_, PNC, and temperature during the study period. About 29% of the PNC measurements and ≤ 2% of all other air pollution and meteorological variables were missing due to a device failure.

### Estimated effects of air pollution

Table 3 shows the percent changes in arithmetic mean values of the ECG parameters [with 95% confidence intervals (CIs)] per interquartile range (IQR; difference between the third and first quartile) increase in PM and PNC. IQR increases in PM levels were associated with significant increases in QTc 24–47 hr later, and borderline significant increases in QTc were associated with 48- to 71-hr lag and 5-day average PM_10_ and PM_2.5_ levels. IQR increases in CO concentrations were also associated with QTc prolongation [0.4% (95% CI, 0.1–0.7%)] 24–74 hr later (data not shown). In general, Tamp was positively associated with air pollution levels 0–23 hr and 24–47 hr before ECG transmission and inversely associated with levels more than 48 hr before, with a significant positive association with PM_2.5_ 0–23 hr before and a significant inverse association with PM_10–2.5_ 96–119 hr before transmission (Table 3). In addition, Tamp was positively associated with NO_2_ 0–23 hr [3.0% (0.2–5.7%)] and 24–47 hr [3.1% (0.1–6.1%)] before transmission. We observed no significant associations between air pollutants and HR (Table 3) or PQ interval (data not shown).

### Estimated effects of air temperature

**Figure 1:**
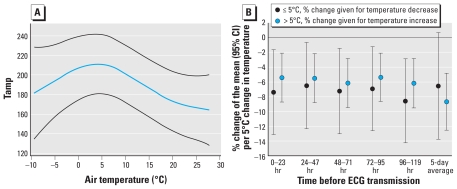
P-spline of air temperature in association with Tamp (*A*) and mean change in Tamp (with 95% CIs) associated with a 5°C increase or decrease in air temperature on days with average air temperature above or below 5°C, respectively (*B*). Both A and B are adjusted for long-term time trend and relative humidity.

HR, QTc, and PQ interval were not associated with temperature changes (data not shown), but we observed an inverse U-shaped association between temperature and Tamp with highest Tamp at 5°C (Figure 1A). To obtain separate temperature effect estimates for warmer and colder days, we modeled temperature as a linear variable and added an interaction term between temperature and a variable indicating mean temperature above or below 5°C. For all lags we observed a 5–9% decrease in Tamp associated with a temperature decline of 5°C on days with average temperatures < 5°C (cold effects) and with a 5°C increase in temperature on days > 5°C (heat effects; Figure 1B).

### Effect modification

**Figure 2:**
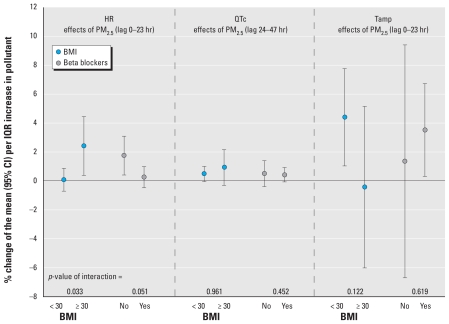
Subgroup-specific associations of IQR increases in PM_2.5_ with ECG parameters (adjusted for long-term time trend and meteorology; PM_2.5_ IQR, 8.4 μg/m^3^).

HR significantly increased in association with an IQR increase in PM_2.5_ 0–23 hr before ECG transmission in individuals with BMI ≥ 30 kg/m^2^ [1.8% (0.4–3.1%)] and in individuals not using beta blocker medication [2.4% (0.4–4.5%); Figure 2]. These effect modifications were significant (*p*-value < 5%) for BMI but only borderline significant for intake of beta blockers. However, only five participants with 200 ECGs did not take beta blockers. No significant interactions between PM and BMI or intake of beta blockers were evident for QTc. Tamp was significantly increased in association with an IQR increase in PM_2.5_ 0–23 hr before ECG transmission in participants with a BMI < 30 kg/m^2^ and in patients taking beta blocker medications. However, the interaction effects were not significant. Additionally, we observed a positive association between Tamp and an IQR increase in CO 24–47 hr before ECG transmission among participants with BMI < 30 kg/m^2^ [2.7% (0.5–4.8%)] and inverse associations with CO among those with BMI ≥ 30 kg/m^2^ [−3.7% (7.4–0.0%)].

We did not evaluate associations with rs18005862 in *HFE* and rs1048943 in *CYP1A1* because MAFs were < 5%. Although rs10183914 in the *NFE2L2* gene was not in HWE, we did not exclude it because our study comprises a highly selected group of MI survivors and not a random population sample. We corrected the global significance level of 5% for testing associations between SNPs and mean levels or variability of ECG parameters for the 8 independent tests resulting in an adjusted alpha-level of 0.05/(8 × 2) = 0.003125. No SNP was significantly associated with mean levels of ECG parameters (data not shown), but some were significantly associated with variability in ECG parameters [see Supplemental Material, Table 2 (doi:10.1289/ehp.1001995)].

**Figure 3:**
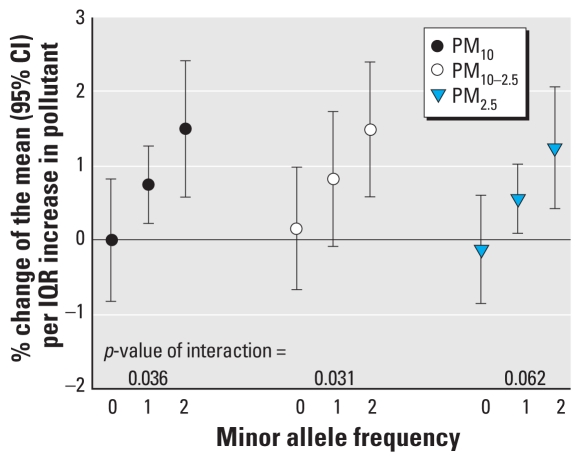
Associations between IQR increases in PM and QTc (lag, 24–47 hr) according to minor allele frequencies of *NFE2L2* rs2364725 (T, wild-type allele; G, minor allele; adjusted for long-term time trend and meteorology; IQRs: PM_10_, 19.5 μg/m^3^; PM_10–2.5_, 10.9 μg/m^3^; PM_2.5_, 8.4 μg/m^3^).

We detected interaction effects between particulate air pollutants and genotypes only for rs2364725 in the *NFE2L2* gene on QTc (Figure 3). Nineteen participants with 363 ECGs were homozygous carriers of the minor allele (G), 28 participants with 885 ECGs were heterozygous, and 20 participants with 497 ECGs were homozygous carriers of the major allele (T). IQR increases in PM 24–47 hr before ECG transmission were associated with a prolonged QTc of about 0.5–1.5% only in patients with at least one minor allele. We also observed a similar pattern for PM exposures 0–23 hr before ECG transmission (data not shown). An IQR increase in PNC exposure 96–119 hr before transmission was inversely associated with QTc in patients with one or two minor alleles [−0.8% (−1.5 to −0.1%) and −2.2% (−3.4 to −1.0%), respectively] but not in other patients [0.6% (−0.4 to 1.6%)].

We detected no other significant air pollution effect modifications with other variables. Temperature effects were not modified by any variable (data not shown).

### Sensitivity analyses

After excluding 10 patients having at least two ECGs with QRS intervals > 120 msec the air pollution associations were in general slightly more pronounced (data not shown). We observed an immediate and 24- to 47-hr lagged increase in HR of about 1% in association with PM. None of the other sensitivity analyses resulted in any notable changes in associations between air pollutants or temperature and the outcomes. We found no evidence for a deviation of linearity of temperature effects on HR or QTc.

## Discussion

Our analyses showed no main effects of air pollutants on HR overall, but we observed significant positive associations between PM 0–23 hr before and HR among participants with BMI ≥ 30 kg/m^2^ and among those not using beta blocker medications. We observed a prolonged QTc interval in association with increases in PM levels 24–47 hr before ECG transmission, with stronger associations among participants with one or two minor alleles of the *NFE2L2* SNP rs2364725. However, patients with at least one minor allele showed shortened QTc in association with an increase in PNC 96–119 hr before. Tamp decreased in association with both cold and warm temperatures, with maximum Tamp around 5°C.

### Air pollutants and ECG parameters

Several authors have reported inverse associations between air pollutants and HRV in the elderly (Luttmann-Gibson et al. 2006; Schwartz et al. 2005). It is hypothesized that air pollutants may activate the sympathetic nervous system directly or indirectly, which possibly leads to an increased HR and reduced HRV. UFP might even translocate into the systemic circulation and affect the electric system of the heart directly (Pope and Dockery 2006). It has been shown that patients not using beta blocker medications exhibit a stronger reduction in HRV in association with PM exposure compared with patients using beta blockers (de Hartog et al. 2009). We observed an increased HR with exposure to air pollutants only among our patients with 200 ECGs not using beta blockers. Beta blockers constrain the activation of the sympathetic tone; thus, participants using beta blockers might be less susceptible to activation of the sympathetic nervous system by air pollutants. Because of the small number of participants not taking beta blockers and even if patient characteristics did not differ significantly between individuals with and without beta blocker intake, it is still possible that the observed differences in the estimated air pollution effects are related to something other than medication intake. Consistent with our findings, Chen et al. (2007) reported stronger positive associations between PM_2.5_ and HR in individuals with BMI ≥ 30 kg/m^2^.

Henneberger et al. (2005) detected an immediate positive association of 24-hr averages of organic carbon with QTc. Furthermore, Liao et al. (2010) reported immediate and delayed QTc prolongations associated with elevated 30-min averages of PM_2.5_. However Lux and Pope (2009) observed no association between PM_2.5_ and repolarization parameters in elderly participants. In our analysis we found lagged associations between PM and QTc. Associations were more pronounced among participants with one or two minor alleles of the *NFE2L2* SNP rs2364725. The *NFE2L2* gene is believed to be involved in the defense against oxidative stress (Goldring et al. 2004). We can only speculate that the defense is more activated in patients with common alleles, whereas patients with at least one minor allele are more susceptible to PM. In contrast, we observed inverse associations between QTc and PNC, a proxy for UFP, in accordance with two chamber studies (Samet et al. 2009; Zareba et al. 2009) that reported QTc shortening in healthy nonsmoking subjects who were exposed to UFP. Different effects of PM and PNC might reflect different biological pathways activated by different particle properties. Tamp indicates the repolarization of the ventricles and Henneberger et al. (2005) reported a 7.3% decrease in Tamp in association with an increase in UFP 0–23 hr before ECG measuring. A subsequent analysis of Yue et al. (2007) suggested that this association was driven by traffic-related UFP specifically. We observed a 4-day delayed decrease and an immediate elevation of Tamp in relation with all PM parameters which cannot be explained by a single patient characteristic such as BMI. It can only be speculated that a combination of medication intake and disease history may be involved in the susceptibility to air pollution. A study by Schneider et al. (2010) also observed opposed variations in Tamp responses depending on the considered PM_2.5_ lag. In general, changes in repolarization might be the result of changes in the ion channel function or a direct effect of the autonomic nervous system on the ventricular myocardium (Ghelfi et al. 2008; Henneberger et al. 2005; Zareba et al. 2009). However, the understanding of the complex biologic pathways is still very limited.

It has been shown that T-wave alternans (TWA) is a reliable predictor for sudden cardiac death (Stein et al. 2010). Zanobetti et al. (2009) observed an association between black carbon and TWA in CAD patients. Prolonged QTc are a risk factor for cardiac arrhythmia (Roden 2008) and cardiovascular mortality (Ziegler et al. 2008). Our results suggest that elevated levels of air pollutants might trigger changes in Tamp and QTc and therefore might predispose to additional cardiovascular problems in individuals who had already experienced an MI.

### Temperature and ECG parameters

Yamamoto et al. (2007) found increased HR and decreased HRV after exposing six healthy Japanese to a heated condition (37°C) in a chamber study. Bruce-Low et al. (2006) observed similar results among participants with ECG measurements before and during exposure to heat in a sauna. In our analyses, HR did not appear to be altered by temperature changes, but during the study period 24-hr averages of temperature never exceeded 28°C, which is similar to the temperature conditions before heat exposure in the previously described studies.

Several studies reported a U- or J-shaped influence of apparent temperature on cardiorespiratory mortality; the lowest mortality was observed for temperatures between 15 and 25°C (Baccini et al. 2008; McMichael et al. 2008). However, Baccini et al. (2008) conducted their study only in the summer, whereas our study took place during almost 1 year with low temperatures during the winter. A study conducted by McMichael et al. (2008) also included Asian and South American cities with high mean temperatures.

Lin et al. (2008) reported an increased risk of cardiovascular events during a follow-up of 30 days among patients with T-wave flattening at the time of the emergency department visit. In our study Tamp decreased with temperature increases as well as decreases. Wolf et al. (2009) observed an inverse relation between temperature and MI occurrence. Therefore, temperature might act as a trigger for T-wave flattening in susceptible individuals, leading to an enhancement of already existing cardiovascular problems.

Because core temperature should not be affected by small changes in ambient temperature, we can only speculate that our observed associations are probably affected by changes in the autonomic nervous system or by loading effects on the heart possibly mediated by cutaneous blood flow regulation or neural input from temperature sensors in the skin.

### Strengths and limitations

A strength of this study is the ability to analyze intraindividual variation because patients transmitted ECG parameters on several occasions. Further strengths are the nonlinear confounder adjustment and the detailed information on patient characteristics and SNPs allowing us to perform several subgroup analyses. Because our estimated effects remained stable in several sensitivity analyses, our results seem to be quite robust. Cardiac arrhythmias alter the interpretation of the ECG parameters; therefore, to have a homogeneous ECG data set, we excluded ECGs with pacemaker rhythms, ventricular trigeminus, or atrial fibrillation. One limitation is that we measured only outdoor exposure, whereas in general people spend a lot of time indoors. However, a study of Cyrys et al. (2004) revealed that ambient concentrations of PM_2.5_ and black smoke can be used as good approximation of indoor concentrations. However, personal measurements of air pollutants should be taken into account in future studies if possible. Because several studies (Analitis et al. 2008; Zanobetti and Schwartz 2008) have shown that changes in temperature are associated with increases in cardiovascular mortality, we assumed that also a short exposure to outdoor temperature possibly influences ECG parameters. A variety of exposure and outcome variables have been used for the analyses, so we cannot exclude that some associations occurred only by chance. A further limitation is that our panel comprised a highly selected group of MI survivors who were taking a variety of medications and might have different reactions to air pollution and temperature compared with healthy people. Thus, generalizability of our results is uncertain. On the other hand, analyzing vulnerable patients might give better insight into possible mechanistic pathways

## Conclusion

Our results indicate that IQR increases in air pollutants were associated with an increase in mean HR among MI patients with BMI ≥ 30 kg/m^3^ and among those not using beta blockers. We observed QTc prolongation in association with an IQR increase in PM in patients with at least one minor allele of a *NFE2L2* SNP. We detected inconsistent associations between air pollution and Tamp, and nonlinear associations between ambient temperature and Tamp. Overall, we observed changes in HR and repolarization parameters associated with air pollutant exposures and temperature changes that are possible precursors for additional cardiovascular problems in individuals who had already experienced an MI.

## Figures and Tables

**Table 1:** Description of the study population of 67 participants with at least one MI.

Clinical characteristic	Mean ± SD or total (%)
Age (years)	59.3 ± 8.5
BMI (kg/m^2^)	29.0 ± 4.3
Systolic blood pressure (mmHg)	127.7 ± 19.9
Diastolic blood pressure (mmHg)	77.7 ± 10.5
Sex (men)	58 (87)
BMI (kg/m^2^)	
< 30	42 (63)
≥ 30	25 (37)
ECG transmission time period	
Winter (October–March)	791 (45)
Summer (April–September)	954 (55)
Type 2 diabetes mellitus	9 (13)
First MI	59 (87)
Angina pectoris	14 (21)
Arrhythmias	15 (22)
Congestive heart failure	6 (9)
Hypertension	28 (42)
Occupational status	
Full-time or part-time employment	29 (43)
Exposed to toxic gases, dust, or fumes during work	15 (22)
Smoking	
Never-smoker	14 (21)
Ex-smoker^a^	53 (79)
Current medication intake	
Beta blockers	62 (93)
Angiotensin-converting enzyme inhibitors	48 (72)
Calcium-channel blockers	8 (12)
Nitrates	9 (13)
Statins	59 (88)
Diuretics	22 (32)
Acetylsalicylic acid	61 (91)
Other antithrombotics	7 (10)

All patient characteristics were determined with a questionnaire during a clinical examination.

a Had stopped smoking at least 3 months before start of the study.

**Table 2:** Description of ECG parameters.

ECG parameter	*n*	Mean ± SD	Minimum	25%	Median	75%	Maximum	IQR
HR (beats/min)	1,745	71.5 ± 11.6	40	63	71	79	114	16
QTc (msec)	1,744	400.6 ± 32.6	270	380	400	420	560	40
Tamp (μV)	1,743	213.4 ± 154.8	−200	100	200	300	700	200
PQ interval (msec)	1,743	164.1 ± 27.8	110	140	160	180	290	40

25% and 75% are 25th and 75th percentiles, respectively.

**Table 3:** Percent change of the outcome mean per IQR increase in pollutant.

	Estimate (95% CI)			
Hr before transmission	PM_10_^a^ (μg/m^3^)	PM_10–2.5_^b^ (μg/m^3^)	PM_2.5_^c^ (μg/m^3^)	PNC^d^ (/cm^3^)
HR				

0–23 hr	0.4 (−0.3 to 1.2)	0.2 (−0.5 to 1.0)	0.5 (−0.2 to 1.2)	−0.4 (−1.2 to 0.5)
24–47 hr	0.5 (−0.3 to 1.3)	0.5 (−0.2 to 1.3)	0.5 (−0.2 to 1.1)	−0.6 (−1.4 to 0.3)
48–71 hr	0.2 (−0.6 to 0.9)	0.1 (−0.6 to 0.9)	0.1 (−0.6 to 0.8)	−0.7 (−1.5 to 0.1)
72–95 hr	0.3 (−0.5 to 1.1)	0.3 (−0.5 to 1.0)	0.2 (−0.5 to 1.0)	−0.4 (−1.2 to 0.5)
96–119 hr	−0.1 (−0.9 to 0.7)	0.1 (−0.7 to 0.9)	−0.2 (−0.9 to 0.6)	−0.5 (−1.5 to 0.4)
5-day average	0.3 (−0.3 to 1.0)	0.4 (−0.3 to 1.1)	0.3 (−0.4 to 1.0)	−1.2 (−2.6 to 0.1)*

QTc				

0–23 hr	0.2 (−0.4 to 0.7)	0.0 (−0.5 to 0.5)	0.3 (−0.2 to 0.8)	0.0 (−0.6 to 0.6)
24–47 hr	0.7 (0.2 to 1.2)**	0.8 (0.3 to 1.3)**	0.5 (0.0 to 1.0)**	0.5 (−0.1 to 1.0)
48–71 hr	0.4 (−0.1 to 0.9)*	0.4 (−0.1 to 0.9)	0.4 (0.0 to 0.9)*	0.2 (−0.4 to 0.8)
72–95 hr	0.3 (−0.2 to 0.8)	0.3 (−0.2 to 0.8)	0.3 (−0.2 to 0.8)	0.1 (−0.5 to 0.7)
96–119 hr	0.3 (−0.2 to 0.8)	0.3 (−0.3 to 0.8)	0.3 (−0.2 to 0.8)	−0.6 (−1.3 to 0.1)
5-day average	0.4 (0.0 to 0.8)*	0.4 (0.0 to 0.8)*	0.4 (0.0 to 0.9)*	0.2 (−0.8 to 1.1)

Tamp				

0–23 hr	3.3 (0.0 to 6.5)*	2.9 (−0.3 to 6.0)*	3.3 (0.2 to 6.3)**	1.7 (−1.7 to 5.1)
24–47 hr	3.0 (−0.4 to 6.4)*	2.5 (−0.8 to 5.8)	2.8 (−0.3 to 5.9)*	2.8 (−0.6 to 6.1)
48–71 hr	−0.8 (−4.1 to 2.4)	−0.8 (−4.0 to 2.3)	−0.5 (−3.5 to 2.5)	−2.4 (−6.1 to 1.3)
72–95 hr	−1.8 (−5.1 to 1.4)	−2.1 (−5.4 to 1.1)	−1.3 (−4.2 to 1.6)	−3.0 (−6.7 to 0.7)
96–119 hr	−2.3 (−5.5 to 0.9)	−3.2 (−6.4 to −0.1)**	−0.9 (−3.8 to 2.0)	−0.8 (−4.6 to 2.9)
5-day average	0.1 (−2.8 to 3.0)	−0.5 (−3.4 to 2.5)	0.8 (−2.3 to 4.0)	−0.7 (−7.0 to 5.6)

a IQR 24-hr average, 19.5 μg/m^3^; IQR 5-day average, 11.9 μg/m^3^

b IQR 24-hr average, 10.9 μg/m^3^; IQR 5-day average, 6.5 μg/m^3^

c IQR 24-hr average, 8.4 μg/m^3^; IQR 5-day average, 6.3 μg/m^3^

d IQR 24-hr average, 7,481/cm^3^; IQR 5-day average, 6,974/cm^3^

* *p* < 0.1.

** *p* < 0.05.
